# The Optical Parameter Optimization for Brain Implant Alzheimer Sensor Using Phototherapy Angle and Wavelength Simulation (PAWS) Methodology

**DOI:** 10.3390/s24227282

**Published:** 2024-11-14

**Authors:** So-Hyun Cho, Chang-Hee Won, Chang-Hyun Kim, Jong-Ha Lee

**Affiliations:** 1 Department of Biomedical Engineering, School of Engineering, Keimyung University, Daegu 42601, Republic of Korea; 1114578@stu.kmu.ac.kr; 2 Department of Electrical Engineering, School of Engineering, Temple University, Philadelphia, PA 19122, USA; cwon@temple.edu; 3 Department of Neurosurgery, Keimyung University Dongsan Hospital, Daegu 42601, Republic of Korea

**Keywords:** Alzheimer sensor, computational simulation, light absorption, phototherapy optimization, therapeutic light angle

## Abstract

Photonic therapy is emerging as a promising method in neuroscience for addressing Alzheimer’s disease (AD). This study uses computational simulations to investigate the impact of specific wavelengths emitted by photodiodes on the light absorption rates in brain tissue for brain implant sensors. Additionally, it presents a novel methodology that enhances light absorption via multi-parameter optimization. By adjusting the angle and wavelength of the incident light, the absorption rate was significantly enhanced using four photodiodes, each emitting at 660 nm with a power input of 3 mW. Notably, an incident angle of 20 degrees optimized light absorption and minimized thermal effects on brain tissue. The findings indicate that photodiodes within the near-infrared spectrum are suitable for low-temperature therapeutic applications in brain tissues, affirming the viability of non-invasive and safe photonic therapy. This research contributes foundational data for advancing brain implant photonic sensor design and therapeutic strategies. Furthermore, it establishes conditions for achieving high light absorption rates with minimal heat generation, identifying optimal parameters for efficient energy transfer.

## 1. Introduction

**Figure 1:**
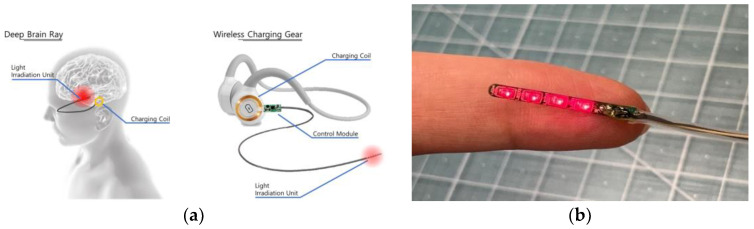
(**a**) Design of an implantable photonic stimulator used for Alzheimer’s disease treatment, (**b**) Implantable photonic device.

In recent years, there has been a notable increase in the application of transcranial phototherapy for Alzheimer’s disease (AD), utilizing red to near-infrared (NIR) wavelengths. This approach is recognized as both novel and safe within the field of neuroscience [1]. As depicted in Figure 1, an implantable photonic stimulator has been developed for AD treatment, designed for deep tissue penetration to stimulate hippocampal neurons with precisely targeted irradiation at 660 nm. This device enhances the expression of brain-derived neurotrophic factor (BDNF) [2]. The device also showed its effectiveness in reducing pain and inflammation has been quantitatively confirmed [3]. Following U.S. Food and Drug Administration (FDA) approval, the implantable photonic stimulator has been widely adopted globally for various therapeutic applications under the name photo biomodulation therapy (PBMT). PBMT converts light energy into cellular energy, thereby activating neurons and producing therapeutic effects, controlled by a sophisticated system that regulates the intensity and duration of light exposure. PBMT converts light energy into cellular energy, thereby activating neurons and producing therapeutic effects, controlled by a sophisticated system that regulates the intensity and duration of light exposure. Although PBMT is primarily employed in treating skin diseases, recent advancements and studies have shown its potential for treating neurodegenerative diseases, including AD. Specifically, PBMT has demonstrated neuroprotective effects in various neurodegenerative disease models [4,5], and has been shown to impact mitochondrial biogenesis in Alzheimer’s disease models [6].

Recent advancements in photonic therapy, particularly using implantable and non-invasive photonic sensors, have demonstrated significant potential in treating neurodegenerative diseases like Alzheimer’s and dementia. Studies have shown that optimizing parameters such as wavelength and power of light sources can enhance the efficacy and safety of these treatments. One notable study employed 660 nm and 850 nm wavelengths with power settings ranging from 3 mW to 5 mW, demonstrating significant improvements in neuronal activity and cognitive functions in Alzheimer’s patients [7,8]. Similarly, just as research using 405 nm and 473 nm wavelengths for optogenetic stimulation has shown precise activation of light-sensitive proteins, PBMT at specific wavelengths, such as 660 nm, can effectively trigger biological responses, highlighting its potential for targeted therapeutic effects. These proteins, such as Channelrhodopsin-2, activate or inhibit neurons in response to light at these wavelengths, allowing for the control of neuronal activity with high spatial precision. This specificity is crucial for mapping neural circuits and manipulating brain functions with minimal impact on surrounding tissues. While this technology is primarily utilized in research contexts and not yet in direct therapeutic applications in humans, it holds significant potential for future clinical use in treating neurological disorders [9,10].

Recent developments in non-invasive techniques have also shown promise. For instance, a study using gamma-wave LED light panels at 40 Hz frequency demonstrated a reduction in brain atrophy and improved cognitive functions in early-stage Alzheimer’s patients [11]. Another investigation utilized 780 nm and 850 nm near-infrared light with power settings between 4 mW and 6 mW, resulting in a significant reduction of amyloid plaques and tau tangles, which are hallmarks of Alzheimer’s disease [12]. Innovative applications of photonic sensors have also been explored in wearable formats. A study highlighted the use of wearable sensors emitting 660 nm red light and 850 nm near-infrared light with low power levels (1–2 mW), showing improved sleep quality and cognitive performance in dementia patients [13]. Furthermore, implantable photonic devices integrated with 3D-printed microfluidic channels using 405 nm and 473 nm wavelengths have been developed to enhance drug delivery and neural stimulation precision [14,15]. Graphene-based implantable electrodes are another groundbreaking advancement, providing high flexibility and low electrical resistance, which ensure efficient neural signal transmission and seamless integration with neural tissues [16].

Additionally, the neuroprotective effects of photo biomodulation therapy have been extensively studied, showing promising results in various neurodegenerative disease models. Near-infrared light therapy has been shown to stimulate mitochondrial biogenesis, potentially mitigating cognitive decline in Alzheimer’s disease models [17]. Quantitative analysis of transcranial and intracerebral light penetration in human cadaver brain tissue provides foundational data crucial for developing computational models. These models help optimize treatment parameters in photodynamic therapy [18]. Studies have demonstrated that LED-based light therapy can reduce cerebral edema and inflammation, improving cognitive functions in neurodegenerative conditions [19].

Our research involves detailed quantitative analysis to optimize the photonic parameters crucial for achieving optimal energy absorption by the targeted tissues. Preliminary results from this study suggest that optimizing certain photonic parameters can significantly influence the efficacy of light-based therapies. Specifically, by adjusting the angle and power settings of a 660 nm photodiode, we observed increased energy absorption within targeted tissues. This adjustment may help in achieving more precise energy delivery while minimizing unwanted heat generation. The incident angle critically influences the depth and dispersion of light as it traverses the CSF to reach the cerebellum. These findings provide essential guidelines for photonic design and application, aiding in the prediction of light trajectory and distribution through cerebrospinal fluid (CSF) and the cerebellum. Additionally, the optimization of energy delivery is a crucial aspect of photonic therapy, aiming to deliver the required energy precisely to the targeted tissue. We explored the range of power settings to establish a threshold where light energy utilization is most efficient while ensuring minimal heat generation, critical for preventing tissue damage. These strategic adjustments to the photonic energy settings and angles are a testament to our commitment to developing a refined approach to photonic therapy that is not only effective but also safer and more adaptable to the needs of individual patients. By precisely controlling the light delivery parameters, our therapy aims to provide targeted.

## 2. Modeling and Method

**Figure 2:**
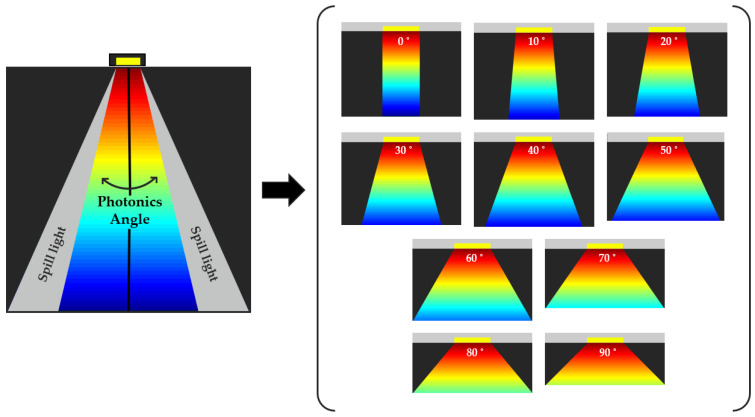
Diagram illustrating the experimental setup for varying the incident angle from 0 to 90 degrees in 10-degree increments. The diffusion of light is visualized as a colormap, with areas of higher intensity represented in red and lower intensity areas in blue.

This study investigates the impact of light wavelength, power, and incident angle on absorption rates in biological tissues in detail. Figure 2 shows the incident angle was systematically varied from 0 to 90 degrees in 10-degree increments to establish optimal conditions. An optimally configured angle enables deeper tissue penetration, ensuring efficient energy delivery to targeted areas. Conversely, suboptimal angles may result in light absorption predominantly at non-targeted locations, reducing therapeutic efficacy. Therefore, precise control and understanding of the incident light angle are imperative to enhance treatment effectiveness. The wavelength of light determines the depth of penetration into tissues and affects the absorption rate. Certain wavelengths penetrate deeper, while others are absorbed more superficially. By optimizing these parameters, more effective and safer photonic treatments can be developed. The methodological modeling in this study involved various experimental setups and theoretical models to examine how changes in angle, wavelength, and power influence absorption rates. The precise control of the incident angle is vital as it directly influences the spread, absorption, and the affected area of the light within the tissue. Variations in the angle of light incidence can significantly alter the degree of light absorption, impacting the efficacy of the treatment. Furthermore, our intention is to employ an invasive approach by creating a small aperture in the skull, into which the device will be inserted and securely anchored using a biocompatible adhesive that is safe for the body. This method ensures that the device remains firmly in place, minimizing any angular deviation during movement. This design consideration significantly reduces the potential for variations in the incident angle, ensuring that the therapeutic light maintains its precise orientation and maximizing the treatment’s effectiveness. By maintaining a stable incident angle, we aim to enhance the precision and predictability of photonic treatments, contributing to improved targeting and treatment of affected brain regions. The optimization of these parameters will play a key role in the development of more effective and safer photonic therapies, as demonstrated through our ongoing research and experimental outcomes.

In this study, we used COMSOL Multiphysics 6.0 software, incorporating a two-dimensional wave optics module. The primary objective was to optimize the light’s absorption rate and energy delivery, facilitated by employing a light source with a 0.3 mm spot size and an analysis of the incident light angle. Through this modeling, the study establishes a framework for evaluating how light reaches the hippocampus and interacts within the brain. Further analysis is conducted on the amount of residual light energy after it passes through the brain layer. This part of the study focuses on the impact of electric field intensity on light absorption and energy propagation. The electric fields discussed in this part of the study refer primarily to those generated by the photodiodes used in our photonic therapy systems. These fields are not from the driving current of the light source, nor are they fields applied specifically to guide the light. Photodiodes in our study generate electric fields as a result of the interaction between the incident light and the photodiode material, which is crucial for the conversion of light into electrical signals. These electric fields directly influence the absorption of light and the subsequent propagation of energy within the device, affecting the overall therapeutic efficacy. Furthermore, the discussion includes an analysis of how the design of the photodiodes and the conditions of light incidence—specifically the angle and power settings of the incident light—were systematically varied in our simulations to evaluate their effects on the intensity and distribution of the electric fields. Our simulations utilized a finite element model to simulate the interaction of light with biological tissues, allowing us to assess how changes in these parameters influence the photobiological effects and therapeutic efficiency. Detailed experimental setups and model configurations will be described in the following sections.

In our simulations, we also evaluated the influence of electromagnetic energy density, which is directly related to its absorption rate within tissues, as a key factor in photonic therapy. The finite element model enabled us to analyze how variations in electromagnetic energy density impact the efficiency of light absorption and, in turn, the therapeutic outcomes. By simulating different energy densities and their distribution within biological tissues, we were able to assess their effects on the photobiological processes. Analyzing energy density at specific points within a tissue allows for detailed comparisons of how light absorption varies across different areas. By pinpointing where energy density is highest or lowest, researchers and clinicians can adjust parameters like light wavelength, intensity, and exposure duration to optimize therapeutic outcomes. This approach not only enhances the precision of treatments but also helps in tailoring them to specific conditions or regions within the body.

### 2.1. Modeling Method

**Figure 3:**
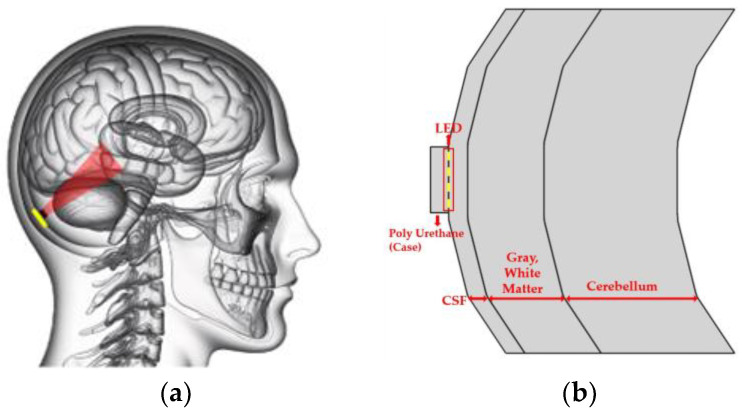
(**a**) Design based on sagittal section of an actual human brain, (**b**) Simulation modeling diagrams.

The model simulates the curved architecture of brain layers such as the CSF, gray matter, and cerebellum, as depicted in Figure 3. In this study, a two-dimensional (2D) model was used instead of a three-dimensional (3D) structure to balance computational efficiency and accuracy. While 3D models can provide detailed geometrical insights, they are computationally intensive, especially when simulating complex biological tissues like the brain. The 2D model allows us to simulate light propagation, absorption, and scattering within tissue layers with reduced computational load, which is particularly useful when examining general trends and behaviors rather than absolute geometrical precision. Although future research incorporating 3D modeling could further refine our findings, the 2D model in this study enabled efficient parameter control and accurate simulation of light–tissue interactions without compromising the quality of the results.

The module’s light-emitting unit is encased in a polyurethane LED case. The dimensions of the case are 16.5 mm in width, 0.8 mm in length, and 0.8 mm in height. Within this setup, the LED array features diodes that are separated by a 1.5 mm gap. We employed a digital brain phantom derived from a high-resolution brain atlas [20] to develop a computational model that simulates the interaction of light in the brain through invasive techniques. Using the Colin27 head atlas, we referenced cross-sectional diagrams from a brain phantom to extract and segment various brain layers, thereby constructing a multi-layered head model [21].

Within this model of the human body’s interior, parameters were meticulously set to account for light transmission, absorption, density, conductivity, extinction, and refractive index. These parameters are essential for depicting the light’s propagation path and optical interactions within the tissue, providing a reliable framework for assessing the light’s trajectory and intensity as it reaches the hippocampus. Further technical details on the modeling parameters and their references are provided in Section 3.2. Through this detailed modeling of light propagation and interaction, the study establishes a framework for evaluating how light reaches the hippocampus and interacts with the brain.

Within the established framework, the absorbed energy density within the tissue is calculated by first simulating the propagation of electromagnetic waves using the set parameters. This involves modeling the light’s interaction with various brain tissues, taking into account factors such as transmission, absorption, and scattering. We analyzed the combined interactions of four LEDs in the system, each emitting light at the same incident angle to ensure comprehensive and uniform light delivery to the target tissue. This approach allowed us to examine how multiple light sources interact with biological tissues simultaneously, affecting light distribution, absorption, and energy density. The electric field distribution within the tissue is then calculated, serving as the foundation for determining energy density at specific points. By examining the spatial distribution of energy density, particularly at key transition points between tissue layers, we can effectively compare the energy density across these regions. This allows for consistent comparisons of how changes in the light source parameters, such as wavelength or intensity, influence energy density. Such analysis is crucial for identifying which variables consistently result in higher or lower energy densities.

The refractive index is a pivotal factor in determining the speed and direction of light within tissues, substantially influencing its propagation and reflection. The scattering coefficient, influenced by the tissue’s microstructure, quantifies the extent of light dispersion within the tissue. Additionally, the absorption coefficient, vital for the conversion of light energy into heat, indicates the degree of light absorption by tissues and varies according to the wavelength [22,23]. This study models the heat and energy transfer within tissues by accounting for variations in the absorption coefficient. Electrical conductivity impacts the transmission of electrical signals and the distribution of heat, while the tissue density affects both light penetration and absorption.

### 2.2. Optical Properties of Modeling

In this study, parameters were meticulously set to account for light transmission, absorption, density, conductivity, extinction, and refractive index within the brain tissues. These parameters are essential for depicting the light’s propagation path and optical interactions within the tissue.

The study’s detailed investigation of the optical properties of key materials and biological tissues, as outlined in Table 1, facilitated a simulation of light’s trajectory through brain tissues [24,25,26]. The specific heat capacity is integral to comprehending how light-induced heat is distributed and the subsequent temperature variations within tissues. Such detailed specifications of complex physical elements are essential for modeling the propagation paths of light and its interactions within tissues. The optical properties of these materials are influenced by factors including the wavelength of light, temperature, composition, and structural integrity.

Table 1 provides the modeled values that reflect actual applications and research, playing a crucial role in optimizing the parameters of photonic therapy and maximizing the effectiveness of optical treatments within brain tissues.

## 3. Light Propagation Modeling Using Finite Element Method (FEM)

The computational simulation conducted in this study utilized an embedded wave optics module based on the finite element method (FEM) to model light propagation.

### 3.1. Geometry

In this study, we did not design the light source itself, but rather adjusted the parameters to suit the chosen beam shape. We employed a Gaussian beam profile to model the light distribution from an LED, specifically chosen to simulate conditions that optimize light absorption by adjusting the angle of incident light. This choice was informed by the LED’s inherent characteristics, including a wide view angle of 120 degrees, which significantly influences its emission pattern. For users adapting our model to alternative light sources, it is crucial to recalibrate the simulation parameters to reflect the specific beam characteristics of their devices. This might involve adjusting the beam shape parameters in the Finite Element Method (FEM) simulations to capture the unique propagation dynamics of light from fiber optics, thereby ensuring that the simulation reflects the light–tissue interaction. Furthermore, for those utilizing fiber optics or other light sources, we recommend validating the modified beam profiles by comparing simulation outputs with experimental measurements of light distribution and absorption. This step is vital to confirm that the model adjustments adequately reflect the physical behavior of light as it interacts with biological tissues.

### 3.2. Optical Properties

Optical properties related to light absorption are determined through interactions, such as absorption and scattering, with various components of biological tissues. Furthermore, the Beer–Lambert law, utilized in this research, describes the attenuation of light as it traverses a material. This formula is derived from the Mie theory, which is a solution of Maxwell’s equations for the scattering of electromagnetic waves by a spherical particle. It is particularly useful when the size of the scattering particles is comparable to the wavelength of the incident light. The formula is essential in understanding how light interacts with small particles, which is applicable in fields such as medical diagnostics (e.g., studying the properties of tissues and cells). The scattering coefficient, *μ_s_*, is determined by factors such as the size, shape, and concentration of particles, as well as the difference in the refractive index between the particles and the surrounding medium. It is calculated using (1):

(1) $$\mu_{s}=\frac{2\pi^{5}\cdot d^{6}}{3\cdot \lambda^{4}}\cdot {\left(n_{particle}-n_{pmedium}\right)}^{2}\cdot N$$

where d denote the thickness of the medium through which the light passes, λ denotes the wavelength of the light,${\;n}_{particle}$ denotes the refractive index of the particle [28], ${\;n}_{pmedium}$ denotes the refractive index of the medium, and *N* denotes the number of particles per unit volume. This formulation illustrates how the degree of scattering within the tissue varies with changes in the wavelength of light.

### 3.3. Simulation Using a Frequency Domain Solver

A Frequency Domain Solver, Maxwell’s equations, was utilized to analyze the state of electromagnetic waves, including light, at specific frequencies. In this study, the frequency domain solver was employed to predict and analyze how electromagnetic waves propagate, reflect, transmit, scatter, and are absorbed.

The interaction between light of specific wavelength and specific power with brain tissue was modeled using the concept of transverse light. Understanding transverse light is crucial for the design of photonic therapies for several reasons:

1. Analysis of Absorption Rates by Wavelength: In our study, we quantitatively analyzed the absorption characteristics of light in brain tissue at specific wavelengths using a frequency domain solver, which calculates absorption rates and predicts how effectively light at these wavelengths can penetrate tissues. Key physical parameters were calculated during the simulations. For example, the scattering coefficient was estimated by analyzing the electric field variations within the tissue, while absorption was determined by evaluating the volumetric heat generation in the tissues. The refractive index was derived using the relationship between permittivity and permeability, providing further accuracy for the simulation. We then analyzed the results using visualization tools to display the electric field distribution, scattering patterns, and energy absorption across the tissue. The data outputs, including scattering and absorption coefficients, were presented in table format, and the total energy absorption was calculated using volume integration.
2. Analysis of the effects of the angle of incident light: In our study, we modeled the interaction of light with brain tissue at varying incident angles using a frequency domain solver. The simulation was designed to explore how different angles affect light absorption and distribution across tissue layers. We tested angles ranging from 0° to 90° in 10-degree increments, examining how each configuration influenced light behavior. We used a Gaussian beam with an emission pattern of 120 degrees, but since our target tissue was the deepest layer, inappropriate angles could lead to uneven light distribution. At steeper angles, energy could be absorbed by non-targeted areas, reducing the effectiveness of light delivery to the intended region. Therefore, we set the maximum angle to 90 degrees to minimize the chances of energy loss in undesired areas and to ensure more controlled light penetration towards the target tissue. This setup allowed us to analyze the impact of varying angles on the distribution and absorption of light, helping us understand which configurations could provide more effective energy delivery to the targeted brain tissue layers.

The transverse field equation, utilized in this study, represents one of Maxwell’s electromagnetic equations. This equation defines the electromagnetic properties of the propagating light and serves as a mathematical model for predicting how electromagnetic waves behave within a medium.

The wave number is a fundamental parameter used to describe the spatial rate of change of light or other electromagnetic waves within a medium, based on frequency and wavelength. It plays a critical role in understanding light propagation in materials and is integral to electromagnetic theories, including Maxwell’s equations. Consequently, the wave number, $k_{0},$ for a given wavelength is used when discussing light propagation in a vacuum, where the refractive index is 1, and there are no external influences on the light wave. $k_{0}$ describes how the wave propagates in a vacuum and is a fundamental parameter used in calculations relating to electromagnetic waves when no medium is present to influence the wave’s properties. It is calculated as follows:

(2) $$k_{0}=\frac{2\pi \cdot \frac{3\;\times \;10^{8}\;[m/s]}{\lambda \;[nm]}}{3\times 10^{8}\;[m/s]}$$

Using a Frequency Domain in simulations provides a quantitative method for modeling and analyzing the propagation characteristics of electromagnetic waves.

### 3.4. Numerical Modeling of Electromagnetic Wave Propagation

Numerical modeling of electromagnetic wave propagation is essential for accurately understanding how signals propagate and reflect within a given medium. To facilitate this, the concept of S-parameters (scattering parameters) is utilized to designate points known as ports, where the incidence and reflection of signals are measured. S-parameters provide crucial information about the locations and characteristics of signal propagation and reflection. This modeling involves designated points known as ports, where the incidence and reflection of these signals are measured.

In this context, Port 1 is defined as the upper boundary where the signal enters the module, and Port 2 is the lower boundary where the signal exits the module after propagation. The S-parameter values measured between these two ports are pivotal in quantifying the energy changes that occur during the signal’s journey. $S_{11}$ and $S_{21}$ are examples of typical S-parameter names, calculated using Port 1 and Port 2, respectively. $S_{11}$ indicates the extent to which some of the propagated light given to Port 1 is reflected to the same Port 1. This parameter measures the extent to which the propagated signal enters the module and is subsequently reflected back to the same port. $S_{21}$ indicates the extent to which the propagated light is transmitted from Port 1 through the module to Port 2. This parameter assesses the effectiveness with which the signal travels through the module from one port to another.

These parameters are defined based on power flow as follows: For Port 1, The propagation constant *k*, derived from classical wave theory, quantifies how the phase of a wave changes as it travels through a medium. The propagation constant (k) is calculated by assigning specific values to the variables in (3). This relationship is pivotal in understanding the interaction of light with materials, specifically how light’s direction and characteristics are modified by refraction and scattering. Refraction occurs when light traverses media with different refractive indices η, altering its speed and wavelength, consequently affecting *k*. Similarly, scattering, influenced by variations in medium properties such as particle size and density, affects how light is redistributed in various directions.

*k* = λ · 2π · η (3)

where λ is the wavelength, *k* is the propagation constant, and η is the refractive index. *k* considers the refractive index of the medium, which alters the wave’s speed and wavelength, resulting in changes in how the wave propagates compared to in a vacuum. *k* is used when light travels through any material medium, where the refractive index *n* is greater than 1.

### 3.5. Simulation of Energy Delivery and Electric Field Analysis

Initially, the simulation assesses the intensity of the electric field (*E*), which serves as an indicator of the directional changes and strength of light as it travels through the tissue. These calculations provide detailed insights into how light intensity is distributed and varies within the tissue, which directly influences the tissue’s rate of light absorption.

Subsequently, electromagnetic energy density (*U*) is calculated. The resulting value is a critical indicator, quantifying both the amount of energy present and the extent of energy loss when light is transmitted through tissue. This information is essential for establishing conditions for the incident light that optimize the absorption and scattering of light energy as it passes through the tissue. The absorption rate of light by biological tissue is another crucial metric. It is calculated using (4):

(4) $$I\left(x\right)=I_{0}e^{-\mu_{a}x}$$

where I(x) represents the light intensity at depth x within the tissue, $I_{0}$ is the initial light intensity upon reaching the tissue surface, and $\mu_{a}x$ is the absorption coefficient of the tissue. This coefficient, which varies depending on the type of tissue and the wavelength of light, indicates the tissue’s efficiency in absorbing light. The parameter x denotes the distance that light propagates within the tissue.

The absorbed energy density, which represents the amount of light energy absorbed by the tissue per unit volume, is calculated using the following equation:

(5) $$u\left(x\right)=\mu_{a}I\left(x\right)$$

where *u* represents the energy absorbed per unit volume, expressed in joules per cubic meter (unit: $J/m^{3}$). Tissues with high absorption rates absorb more light energy, which can either amplify therapeutic effects or induce thermal damage at specific depths.

### 3.6. Modeling Heat Transfer in Brain Tissue Using Light

In this section, we apply Fourier’s law to develop a computational model that quantifies heat transfer phenomena within the brain’s complex biological tissues. This model is critical for predicting the thermal effects induced by photonic stimulation, a key concern in neurological therapies.

The model incorporates heat conduction equations tailored to the unique properties of brain tissue, accounting for both the diffusive transfer of heat and the localized absorption due to external light sources. Specifically, we use the equation q=−k∇T, where q represents the heat flux, k the thermal conductivity of the tissue, and ∇T the temperature gradient within the tissue. This formulation allows us to simulate how rapidly and extensively heat spreads following photonic exposure.

Our simulations consider variations in thermal conductivity values reported in the literature for different types of brain tissues [29,30]. Moreover, we model both internal metabolic heat generation and external heat input from light sources, thus providing a comprehensive view of the thermal landscape under photonic influence. We present a series of simulations under various operational conditions, demonstrating how changes in light intensity, wavelength, and exposure duration affect the brain tissue’s thermal response. The results are quantitatively analyzed, showing specific thresholds of thermal change that are crucial for safe therapeutic applications. The model’s predictions are validated against experimental data from peer-reviewed sources that detail similar experimental setups [31]. This validation confirms our model’s accuracy in predicting thermal responses, underscoring its potential utility in designing safer, more effective photonic therapies for neurological disorders. By refining our model based on Fourier’s law and focusing on quantitatively simulations, we offer detailed insights into the heat transfer mechanisms within brain tissues.

## 4. Experiment Results

**Figure 4:**
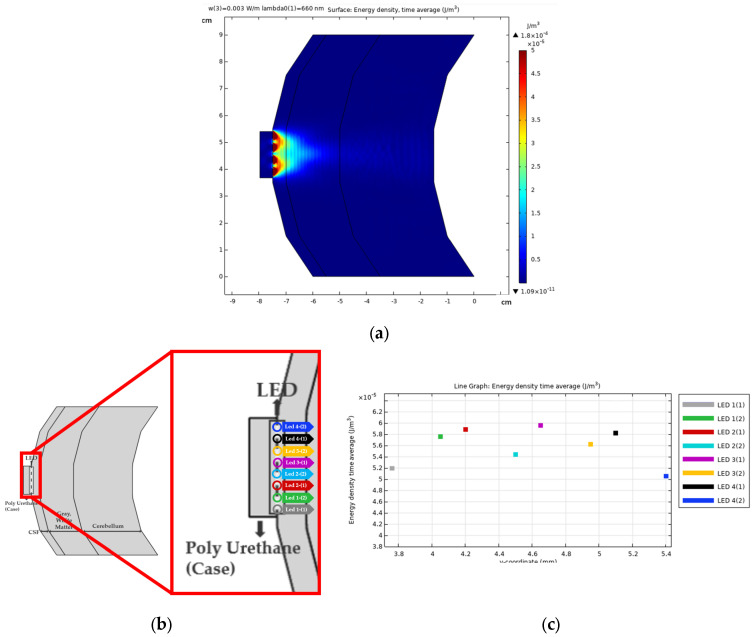
Diagram showing the distribution of energy from the LED component: (**a**) Results of the overall, (**b**) Perspective of photonics, (**c**) Frontal view of photonics.

Figure 4 presents a cross-sectional diagram illustrating the distribution of light energy emitted by the four photodiodes implanted in the brain. Photodiodes 1 and 4, positioned at the edges of the device, are implanted intracranially. They are implanted within the brain tissue through small apertures drilled in the skull. The placement of 1-(1) and 4-(2) is such that one side of each photodiode interfaces directly with the photodiode casing rather than another photodiode. This reduces the amount of light overlap compared to photodiodes 2 and 3. As a result, 1-(1) and 4-(2) transmit lower energy. Photodiodes centrally located between 1-(2) and 4-(1) benefit from mutual light diffusion. The polyurethane casing, chosen for its low light absorption properties, further directs light away from the skin and towards the cerebrospinal fluid (CSF), enhancing light transmission to the target region.

The use of polyurethane in our design was a deliberate choice to improve the overall optical performance of the device. Polyurethane is known for its low light absorption rate, which ensures that minimal light is absorbed by the casing itself. This property allows a greater amount of light to be directed into the brain tissue, enhancing both the amount and the directionality of light that reaches the CSF and surrounding tissues. The material minimizes unnecessary energy loss and supports the precise targeting of therapeutic light in the brain. For reference, studies on polyurethane’s optical properties demonstrate its suitability for applications requiring low light absorption and high transmittance, such as optical devices [32]. These features make it an ideal material for use in devices that rely on controlled light delivery, like the photodiodes in our design.

**Figure 5:**
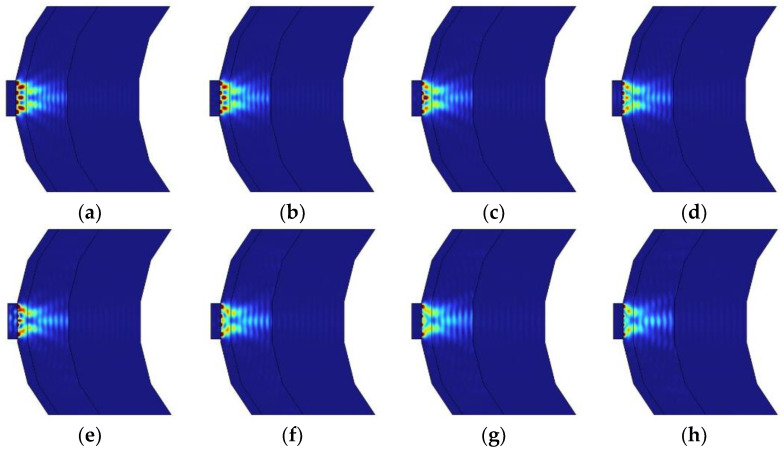
Absorption rates of brain tissue across different wavelengths displayed on a 2D surface. The red areas indicate regions of high energy absorption by the medium: (**a**) 660 nm, (**b**) 690 nm, (**c**) 720 nm, (**d**) 750 nm, (**e**) 780 nm, (**f**) 810 nm, (**g**) 840 nm, (**h**) 870 nm.

**Figure 6:**
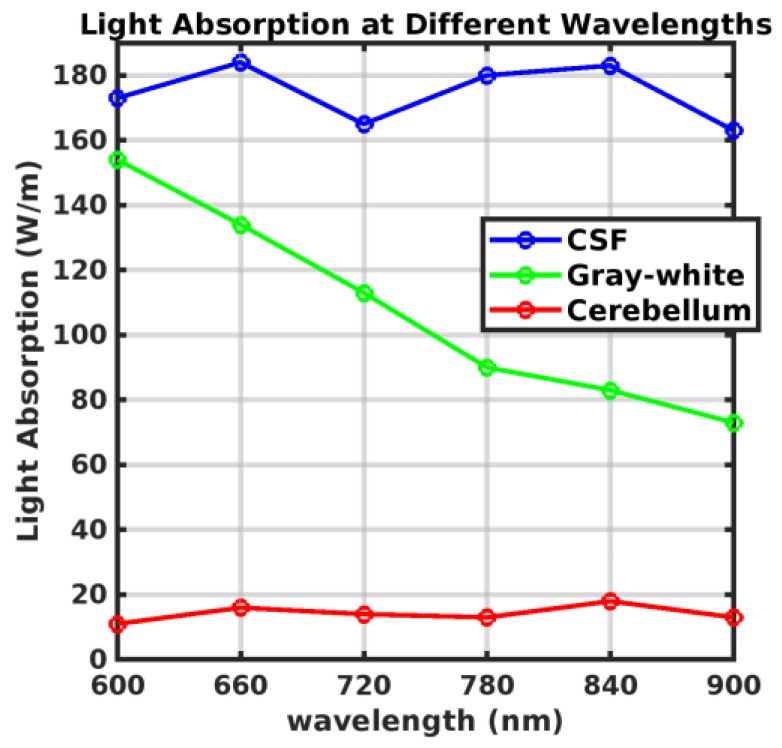
Graphical representation of varying light absorption rates across different wavelengths.

Figure 5 illustrates the light absorption rates of brain tissue at different wavelengths on a 2D surface. Subsequent graphs in Figure 6 plot light absorption rates as they vary with wavelength. The red areas in the graph can be interpreted as regions where the medium absorbs a high amount of energy. This indicates that these sections are where the light absorption rate is significantly elevated. This data is crucial for determining the optimal conditions for phototherapy at specific wavelengths, as it quantitatively demonstrates how different types of brain tissue absorb light across the spectrum. 

According to the data presented in Figure 6, two distinct minor peaks were observed at 660 nm and 840 nm for the light absorption rates in the cerebellum. These measurements highlight that the maximum absorption rates were distinctly observed at specific wavelengths. This finding suggests that these specific wavelengths could be particularly beneficial in the development of phototherapy devices. The continuous increase in absorption observed in gray matter relates to its optical properties. Gray matter, being rich in neuronal cells, contains chromophores that are optically active. These chromophores absorb light more effectively at increasing wavelengths. Moreover, gray matter is vascular-rich, containing additional optical absorbers like hemoglobin in the blood. These absorbers increasingly effectively absorb light as the wavelength extends into the red and near-infrared spectrum. Therefore, the gray matter has a higher absorption coefficient than other tissue layers.

Recent studies, such as those conducted by Hamblin [9], have underscored the effectiveness of specific wavelengths in photo biomodulation, particularly highlighting the dual absorption peaks of cytochrome c oxidase (CCO) near 830 nm and 665 nm. These findings provide a robust foundation for our choice of 660 nm in the development of our phototherapy device. Although the absorption at 830 nm is significant, their research suggests that 660 nm offers superior tissue penetration and reduced light scattering, enhancing energy delivery to deeper brain tissues. In our study, we observed distinct absorption peaks at both 660 nm and 840 nm in the cerebellum, supporting the potential of these wavelengths in therapeutic applications. However, the superior penetration capabilities of 660 nm, as suggested by the literature, led us to hypothesize that this wavelength would be more efficient for our specific therapeutic needs. To validate this hypothesis, we conducted additional simulations with different parameters to rigorously compare the two wavelengths.

**Figure 7:**
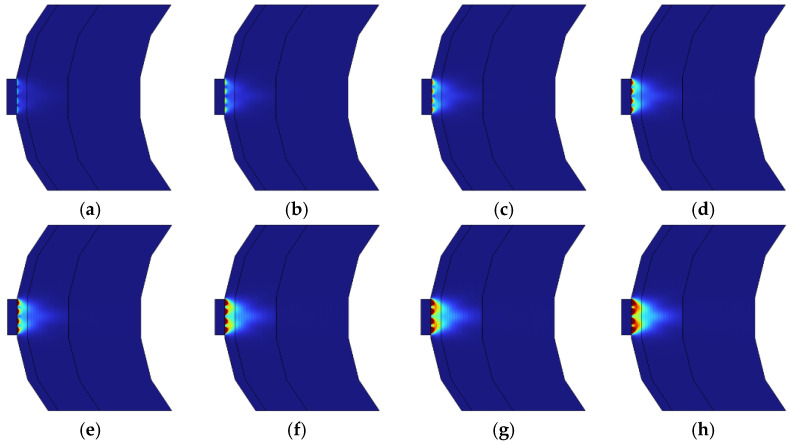
Illustration of how light absorption rates in brain tissue increase with power on a 2D surface. The red areas indicate regions of high energy absorption by the medium.: (**a**) 1 W, (**b**) 2 W, (**c**) 3 W, (**d**) 4 W, (**e**) 5 W, (**f**) 6 W, (**g**) 7 W, (**h**) 8 W.

**Figure 8:**
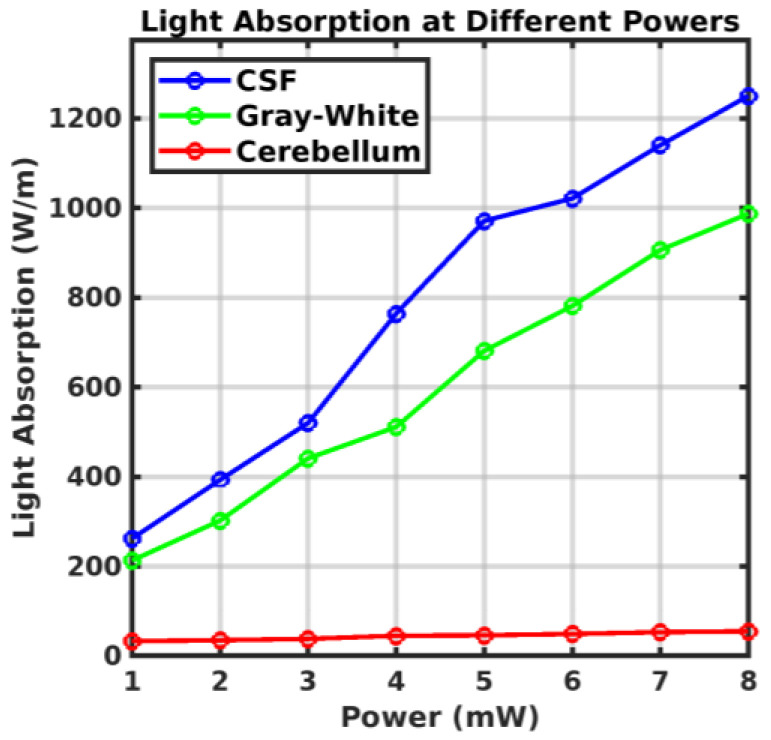
Graphs depicting the changes in light absorption rates as power increases.

Figure 7 illustrates the light absorption rates of brain tissue at different power on a 2D surface. In our study, the term “light absorption rate” refers to the ratio of light absorbed by a medium to the total light that reaches the medium. This rate is typically expressed as the amount of energy absorbed by the medium at a specific wavelength divided by the total energy that reached the medium. The light absorption rate is calculated as the ratio of the total energy of light that reaches a medium to the energy absorbed by the medium. For instance, when light is shone on a medium of a specific thickness, the light absorption rate can be measured by comparing the energy absorbed to the initial energy. The light absorption rate is a critical parameter for assessing optical properties and understanding the optical responses of materials. This parameter is essential in fields such as phototherapy, optical sensors, and solar panels, where optimizing the light absorption rate of materials can significantly enhance efficiency. Figure 8 plots light absorption rates as they vary with power. As depicted, the light absorption rates all proportionally increase with enhanced power. This is an expected outcome, as more energetic photons typically enhance light absorption.

**Figure 9:**
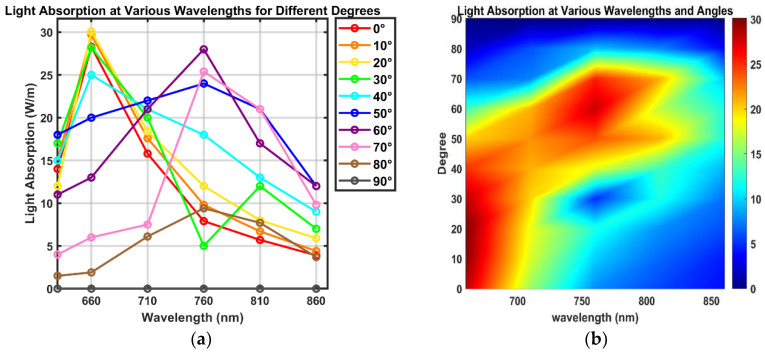
Analysis of how light absorption rates vary with incident angle and wavelength: (**a**) Line graph, (**b**) 2D graph with color map.

Due to the curved shape of the human head, we determined that the absorption rate of light would vary depending on the angle of incident light. Therefore, we conducted additional simulations focusing on this angle. Through these simulations, adjusting the angle of incident light was identified as a critical parameter for maximizing the effectiveness of photonic therapy, significantly affecting absorption rates. Figure 9 provides a detailed analysis of how changes in the incident angle of the photodiode affect light absorption rates. The data shows that the maximum absorption rate reaches 30.1 W/m at a wavelength of 660 nm and an angle of 20 degrees, and 18.4 W/m at 840 nm and an angle of 50 degrees. This indicates that 660 nm could maximize absorption rates in the cerebellum, providing crucial evidence for determining the most effective energy delivery angles for therapeutic applications.

**Figure 10:**
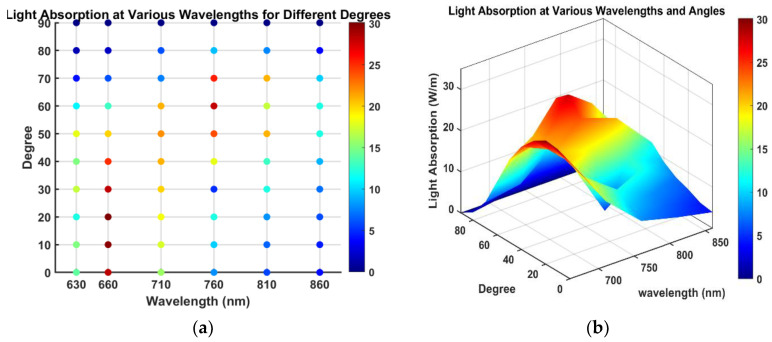
Comprehensive analysis of light absorption rates influenced by changes in incident angle and wavelength: (**a**) Dot plot with color map, (**b**) 3D graph with color map.

Figure 10 presents 3D graphs illustrating the comprehensive effects of incident angle and wavelength on light absorption rates, highlighting the complex interactions between these variables. Notably, the highest absorption rates were observed at 660 nm and 20 degrees, consistent with findings from 2D graphs.

**Figure 11:**
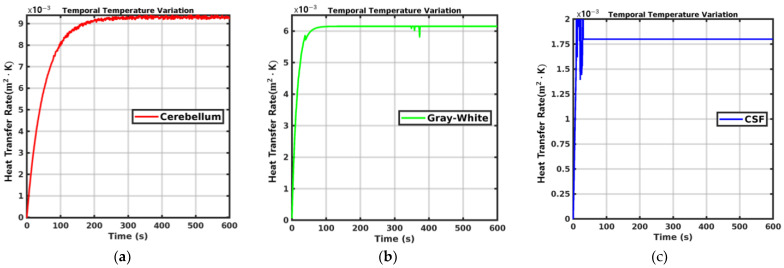
Temporal variations in temperature across different brain tissues: (**a**) cerebellum, (**b**) gray–white matter, and (**c**) CSF.

Figure 11 presents a detailed comparison of heat transfer dynamics across three brain tissues: the cerebellum, gray–white matter, and cerebrospinal fluid (CSF), depicted in panels (a), (b), and (c), respectively. The *x*-axis represents time in seconds, and the *y*-axis represents the heat transfer rate. Cerebellum (a) shows a rapid increase in heat transfer rate, peaking within the first 100 s, followed by a plateau, indicating a quick reach to thermal equilibrium. This suggests an initial high thermal conductivity in the cerebellum, which quickly stabilizes, reflecting its ability to efficiently absorb and dissipate heat due to metabolic activities. Gray–White Matter (b) maintains a consistent heat transfer rate, indicative of stable thermal properties and minimal fluctuations throughout the period observed. This uniformity suggests homogenous thermal characteristics, likely due to similar cellular structures and water content within this tissue type. CSF (c), in stark contrast, exhibits a significantly lower and more stable heat transfer rate, underscoring its role as a thermal insulator. The CSF’s high water content, which inherently possesses a high specific heat capacity, requires more energy to change its temperature compared to tissues with lower water content, thereby moderating rapid temperature changes. Furthermore, the continual circulation of CSF in the subarachnoid space and ventricles facilitates heat exchange with surrounding tissues, aiding in maintaining a constant temperature around the brain and spinal cord.

The simulations presented in Figure 11 were conducted across a range of power levels, from 1 mW to 9 mW, and the resulting temperature profiles were remarkably consistent across all tested conditions. The graph showed minimal fluctuations in temperature, with values consistently in the range of $10^{-3}$, regardless of the power level applied. This uniformity in the data demonstrates that the changes in power did not lead to any significant temperature variation in the tissues, ensuring the findings remain valid for all power settings. The results confirm that even at higher power levels, there was no notable increase in temperature that could lead to tissue damage, further supporting the thermal safety of the procedure.

According to the referenced temperature change rate, it can be observed that the cerebellum exhibits no significant deviation from the initial starting temperature at a change rate below 0.01 $m^{2}\cdot K$. This demonstrates the efficient thermal regulation attributed to the simulated general heat distribution properties of the cerebellar tissue, rather than direct modeling of blood vessels. This demonstrates the thermal stability of cerebellar tissue, providing a critical reference point to ensure safe and effective phototherapy protocols. These datasets provide essential guidelines for temperature management when using LED light sources in clinical settings or biomedical research.

## 5. Result

This study has identified key factors in optimizing phototherapy, including placement, photonic case material, wavelength, power, and particularly the angle of incident light. By utilizing a 660 nm photodiode with power settings between 3 and 4 mW, we found that a precise 20° angle of incident light maximizes absorption and minimizes scattering in the cerebrospinal fluid and cerebellum. Optics computer simulations modeled these interactions, showing an increase in absorption rates within this power range, pointing to optimal conditions for efficient light energy use with minimal heat generation. This approach underscores the critical role of precise control over the incident light angle to maximize therapeutic effects and ensure effective energy delivery to deeper tissues. Our experimental results further confirm that these variables significantly influence the amount of light delivered to tissues, directly impacting phototherapy’s effectiveness. Notably, the high absorption rate at 660 nm showcases its potential for brain tissue therapy. Achieving maximum absorption at a 20° angle underscores the importance of angle adjustments in phototherapy design, while low-temperature variations indicate that the light sources operate within safe limits, reducing the risk of thermal damage.

**Figure 12:**
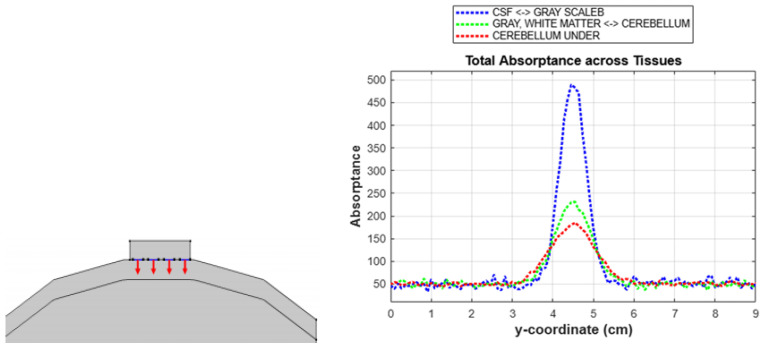
Mesh independence test; The arrows indicate the direction of light injection.

**Figure 13:**
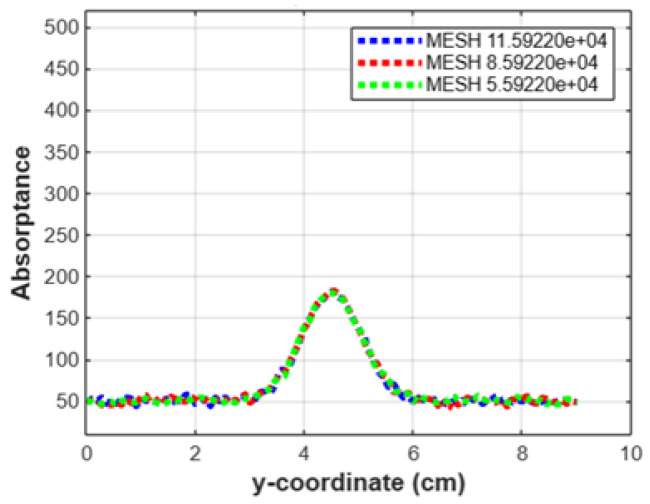
Mesh independence test.

Additionally, we performed a thorough analysis of absorption levels across different tissue layers, as displayed in Figure 12. The *x*-axis of the graph represents the horizontal length of the model, and the *y*-axis indicates the total absorptance. This approach allowed us to assess light absorption at specific points along the model and better understand how light interacts with various tissues. To further confirm mesh independence, we conducted a focused study on the cerebellum, the lowest layer in our model. As shown in Figure 13, we conducted a detailed mesh independence test to ensure the robustness of our simulation results. By comparing the absorption rates across fine, moderate, and coarse mesh configurations, we observed that the values remained consistent regardless of mesh density. This consistency demonstrates that refining or coarsening the mesh does not impact the simulation accuracy, confirming that our model is mesh independent. These findings validate the sufficiency of the chosen mesh density for reliable data collection, negating the need for further refinement to maintain result accuracy.

**Figure 14:**
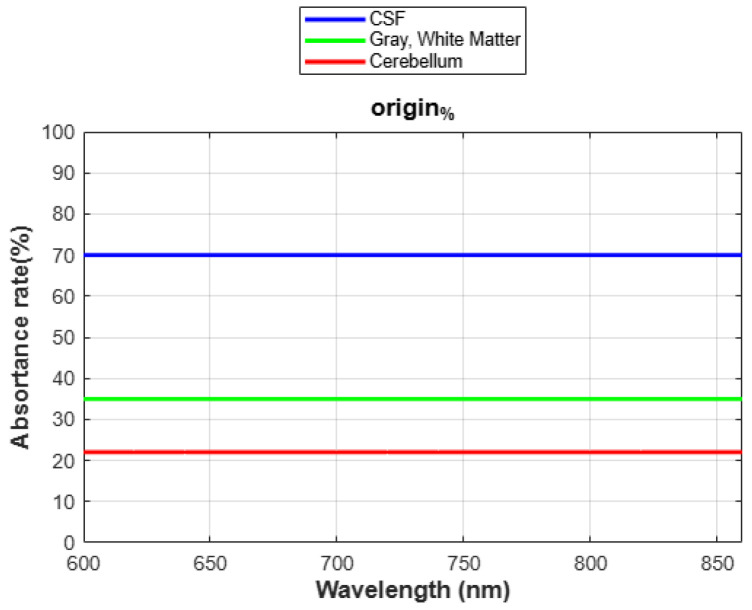
Absorption and Scattering Coefficients Not Considered with Respect to Wavelength.

**Figure 15:**
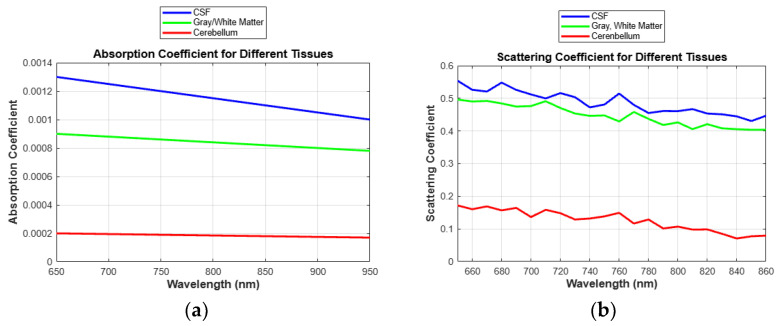
(**a**) Absorption Coefficient of Tissue as a Function of Wavelength, (**b**) Scattering Coefficient of Tissue as a Function of Wavelength.

Initially, our analysis did not account for variations in absorption and scattering coefficients across different wavelengths, resulting in uniform absorption levels across all wavelengths, as shown in Figure 14. This uniformity stemmed from simplifying assumptions of constant coefficients, which inaccurately represented light–tissue interaction. Our findings stress the need to incorporate wavelength-dependent variability to produce more accurate and realistic simulations. By refining our model, we successfully simulated complex light–tissue interactions, a critical step for effective PBMT. The angle of incidence was calculated using the data from Figure 15, organized into Excel sheets for detailed analysis. Adjustments were then made to compare resulting absorption rates, as demonstrated in Figure 10. Furthermore, our discussion emphasizes that precise parameter control extends beyond absorption coefficients and includes factors like the angle of incidence. Controlling these variables enabled us to map absorption levels at specific points along the *x*-axis of our model, providing a comprehensive understanding of how different regions within the tissue absorb light. This refined approach allows for more effective optimization of phototherapy treatments.

By optimizing light absorption in specific tissues, treatment efficiency can be improved while minimizing the risks of overheating and other side effects. These findings open avenues for further research on various tissue types and pathological conditions, expanding the scope and effectiveness of phototherapy. They also provide a deeper understanding of the optical properties of brain tissue, aiding in the development of personalized strategies for enhanced therapeutic outcomes. Moving forward, it is essential to continue refining PBMT for broader applications. Future research should explore a wider range of optical parameters across different wavelengths, power settings, and tissue depths. Additionally, incorporating temperature control mechanisms and adaptive feedback systems to monitor real-time tissue responses will help ensure safer and more effective treatments. These innovations could extend the clinical applications of phototherapy, broadening its therapeutic potential beyond brain-related conditions to other medical treatments.

## Figures and Tables

**Table 1:** Detailed results from the absorption spectra analysis [27].

Purpose	Poly Urethane	CSF	Gray, White Matter	Cerebellum
Diameter (mm)	0.8	0.5	2.5	3.5
Refractive index, real part	1.6	1.33	1.5	1.42
Absorption Coefficient (1/m)	0.0001	0.0013	0.0009	0.0002
Scattering Coefficient (1/m)	0.5	0.01	0.05	0.1
Conductivity (W/(m·k))	0.15	0.5	0.5	0.51
Density (kg/m^3^)	1.1	1007	1045	1045
Heat Capacity (J/(kg·k))	1800	3850	3500	3653

## Data Availability

The data that support the findings of this study are available from the corresponding authors upon reasonable request.
